# Evaluation of classification and forecasting methods on time series gene expression data

**DOI:** 10.1371/journal.pone.0241686

**Published:** 2020-11-06

**Authors:** Nafis Irtiza Tripto, Mohimenul Kabir, Md. Shamsuzzoha Bayzid, Atif Rahman

**Affiliations:** Department of Computer Science and Engineering, Bangladesh University of Engineering & Technology, Dhaka, Bangladesh; Polytechnical Universidad de Madrid, SPAIN

## Abstract

Time series gene expression data is widely used to study different dynamic biological processes. Although gene expression datasets share many of the characteristics of time series data from other domains, most of the analyses in this field do not fully leverage the time-ordered nature of the data and focus on clustering the genes based on their expression values. Other domains, such as financial stock and weather prediction, utilize time series data for forecasting purposes. Moreover, many studies have been conducted to classify generic time series data based on trend, seasonality, and other patterns. Therefore, an assessment of these approaches on gene expression data would be of great interest to evaluate their adequacy in this domain. Here, we perform a comprehensive evaluation of different traditional unsupervised and supervised machine learning approaches as well as deep learning based techniques for time series gene expression classification and forecasting on five real datasets. In addition, we propose deep learning based methods for both classification and forecasting, and compare their performances with the state-of-the-art methods. We find that deep learning based methods generally outperform traditional approaches for time series classification. Experiments also suggest that supervised classification on gene expression is more effective than clustering when labels are available. In time series gene expression forecasting, we observe that an autoregressive statistical approach has the best performance for short term forecasting, whereas deep learning based methods are better suited for long term forecasting.

## Introduction

Microarray time series gene expression experiments have essential applications in studying cell cycle development [1, 2], immune response [3], and other biological processes. Monitoring the change in gene expression patterns over time provides opportunities to study mechanistic characteristics of various cellular processes. The Stanford Microarray Database (SMD) [3] stores raw and normalized data from microarray experiments and provides web interfaces for researchers to retrieve, analyze, and visualize their data. Analyzing time series gene expression data has various significance, such as genetic interaction and knockout screens, understanding of development, cellular response to drug treatment [4], tumorigenesis [4], infection or disease identification, and determining correlated genes [5]. However, existing studies mostly utilize gene expression values for clustering gene profiles and rarely focus on performing tasks such as classification, forecasting or anomaly detection [6].

Time series data analysis is widely used in various domains such as financial [7], weather prediction [8], forecasting of diseases [9], and therefore, different techniques have been developed for their analysis. For example, we can analyze the time value of money or demand for a product in the future from time series prediction. Weather forecasting and humidity/temperature prediction [8] also have significant implications. Classification on time series also allows us to isolate noise and identify irregular patterns from trend and seasonal data points [10, 11].

Gene expression data obtained from microarrays or RNA-seq are often arranged in a time-ordered fashion in a gene expression matrix (GEM), with genes in rows and experiments in columns. In each row, there is a time series that indicates the expression of a particular gene at different time points. Each column corresponds to a microarray experiment that contains the expression values of all genes for that particular time or condition. Throughout the paper, we utilize the term gene expression/profile or time series to indicate a row in the gene expression matrix. Sometimes, there are labels associated with gene expression profiles. Existing works in microarray gene expression matrices either focus on clustering gene expression profiles (rows) or classification of experiments array (columns). However, in this study, we concentrate on gene expression classification and forecasting problems, which have rarely been studied in the existing literature. By classification, we refer to classifying gene expression profiles (rows) according to their labels. Forecasting tries to predict expression values for each gene from existing time points. Both of them have significant applications. Classification can distinguish anomalous time series and thereby remove noise from gene expression. Moreover, it may allow us to inspect the behaviour of the cell cycle or to detect specific genes associated with disease progression. Forecasting can identify missing data points in gene expression data and predict the behaviour of a specific gene in future time points where experimental values are not available.

Several clustering methods have been studied to analyze these gene expression data and use these clusters to identify gene groups. These include popular clustering techniques such as hierarchical clustering [12], k-means clustering [13], and self-organizing maps [14]. Ernst et al. [15] presented an algorithm specifically designed for clustering short time series expression data. Their algorithm works by assigning genes to a predefined set of model profiles that capture the distinct potential patterns that can be expected from the experiment. A software STEM [16] has also been implemented to provide an interface for different short time series clustering and visualization. There also have been several studies regarding the challenges in gene expression clustering, such as missing values in gene expression [17], unequal time interval, and an unequal number of time points in various gene expressions [18]. However, the choice of clustering procedure, including the proximity measure, has a tremendous impact on the gene clustering. The attributes available are a direct consequence of the experiment that was conducted, and the intended gene clustering based on a time-course experiment may differ from the clustering obtained [19]. Given the lack of natural gene clusters, many datasets currently subjected to cluster analysis would yield more informative results, if approached with methods for supervised learning [20]. Therefore, utilizing gene expression phase or label could increase the validity and performance of gene expression grouping [21, 22].

However, present studies in the microarray gene expression classification are mostly designed to classify experiment samples (columns in the gene expression matrix), for example, the difference between cancerous gene expression in tumour cells and the gene expression in normal, non-cancerous tissues. Gene expression matrix is typically very narrow, i.e. the number of genes is significantly larger than the number of experiments [23]. Current works focus on different feature subset selection procedures, such as Genetic algorithm [24], Information gain [25], recursive feature elimination [26] and then apply unsupervised or supervised machine learning approaches for classification [27]. Therefore, these methods do not apply to our study since we aim to classify gene expression profiles (rows) that only have a few features.

There is a limited number of prior studies in the literature that focus on gene expression classification. Lin et al. [28] utilized Hidden Markov Models (HMM) with fewer states than time points leading to an alignment of gene expression from different patient response rates and proposed this discriminative HMM for classification. Orsenigo and Vercellis suggested a temporal extension of L1-norm Support Vector Machines (SVM) that uses dynamic time warping distance for measuring time series similarity [29]. Cui et al. presented a classification of human circadian genes based on time-course gene expression profiles using deep neural networks (DNN) [30]. They transformed time series into categorical-state data to denote the changing trend of gene expression and then applied DNN to discriminate between aperiodic and two subclasses of periodic genes. Recently Ozrul et al. [31] proposed a new framework DeepTrust that initially transforms time series data into images to obtain richer data representations and later employs a deep convolutional clustering algorithm on the constructed images. They evaluated their approach in a biological and simulated dataset with short time series.

While time series forecasting is a major area of research, there have been very few studies specifically on forecasting of time series gene expression data. Prior works on gene expression inference try to predict expression profiles (complete rows in gene expression matrix) of target genes (test samples) from landmark gene expression profiles (training samples) [32–34]. But we focus on predicting future expression values of genes from existing values for all genes. In other domains, however, many statistical and machine learning-based methods have been developed for this purpose. ARIMA (Autoregressive Integrated Moving Average) [35] and Holt-Winters (Triple Exponential Smoothing) [36] are the two most popular and widely used statistical forecasting methods utilized in various domains. While the Holt-Winters model is based on a description of trend and seasonality in the data, ARIMA aims to describe the correlations in the data. As a machine learning model, Kim applied SVM to predict the stock price index from previous values [37]. Qiu et al. [38] proposed an ensemble of deep learning belief networks (DBN) for regression and time series forecasting and aggregated the outputs from various DBNs by a support vector regression (SVR) model. Later, Kuremoto et al. [39] presented a method for time series prediction using Hinton and Salakhutdinov’s deep belief nets (DBN) which are probabilistic generative neural network composed of multiple layers of restricted Boltzmann. Prophet is also a popular forecasting procedure, recently developed by Facebook that works based on an additive model and performs best with time series that have strong seasonal effects and several seasons of historical data [40]. Therefore, it is not applicable for time series gene expression. Recently, Alexandrov et al. [41] developed Gluon Time Series (GluonTS), a toolkit for probabilistic time series modelling, focusing on deep learning based models. It includes different generative, discriminative, and auto-regressive models and can be applied to time series from various domains. However, the applicability of these forecasting methods in gene expression domain is yet to be thoroughly investigated in the present literature.

In this study, we assess statistical, machine learning and deep learning based methods for time series gene expression data forecasting and classification through an extensive experimental study on five real gene expression datasets. We present novel Convolutional Neural Network (CNN) and Long Short Term Memory (LSTM) based methods for time series classification and compare their performances with deep learning based approaches DNN and DeepTrust as well as state-of-the-art traditional methods for classification. To illustrate why classification is more meaningful than clustering when labels are available, we also perform clustering on these datasets using the STEM software since it is widely used for clustering short time series gene expressions [16].

For forecasting, we evaluate the popular Holt-Winters and ARIMA methods as well as the Feed Forward Neural Network [42] from the GluonTS toolkit. In addition, we implement an Artificial Neural Network (ANN), and LSTM models for gene expression forecasting and include them in the comparison. We vary the periods for which expression is to be forecast to reveal the methods suited for short and long term forecasting.

## Materials and methods

In this section, we provide a detailed description of the classification and forecasting approaches we have evaluated. We also discuss the datasets used in our study and describe the evaluation criteria for performance analysis.

### Classification

Given a set of gene expression *G* = {*g*_*i*_} where each expression *g*_*i*_ denotes a time series of *T* time periods of the *i*-th gene as $g_{i}=\{x_{i_{1}},x_{i_{2}},x_{i_{3}},\ldots ,x_{i_{T}}$} and $x_{i_{t}}$ denotes the expression level at the *t*-th timepoint, the classification problem tries to predict the label or class of the gene *g*_*i*_ based on its expression values and already predicted gene expressions. Formally, let (*g*_*i*_, *c*_*i*_) be a training instance with *T* time points $x_{i_{1}},x_{i_{2}},x_{i_{3}},\ldots ,x_{i_{T}}\equiv g_{i}$ and a discrete class variable *C* which takes some finite discrete values (i.e., one of the possible classes). A dataset *D* is a set of *n* such training instances: *D* = {(*g*_1_, *c*_1_), …, (*g*_*n*_, *c*_*n*_))}. The classification task consists of learning a classifier on *D* such that any additional gene expression *g*′ can be classified based on the expression value of *g*′ and gene expressions in *D*.

In this study, we are performing classification on labelled datasets where each gene belongs to a specific class and consider each expression to consist of the same number of time points. The methods for classification evaluated in this study are described below where the CNN and LSTM architectures are proposed in this paper, and SVM, One-Class SVM, DNN [30] and DeepTrust [31] are from the literature.

#### Convolutional Neural Network (CNN)

**Fig 1:**
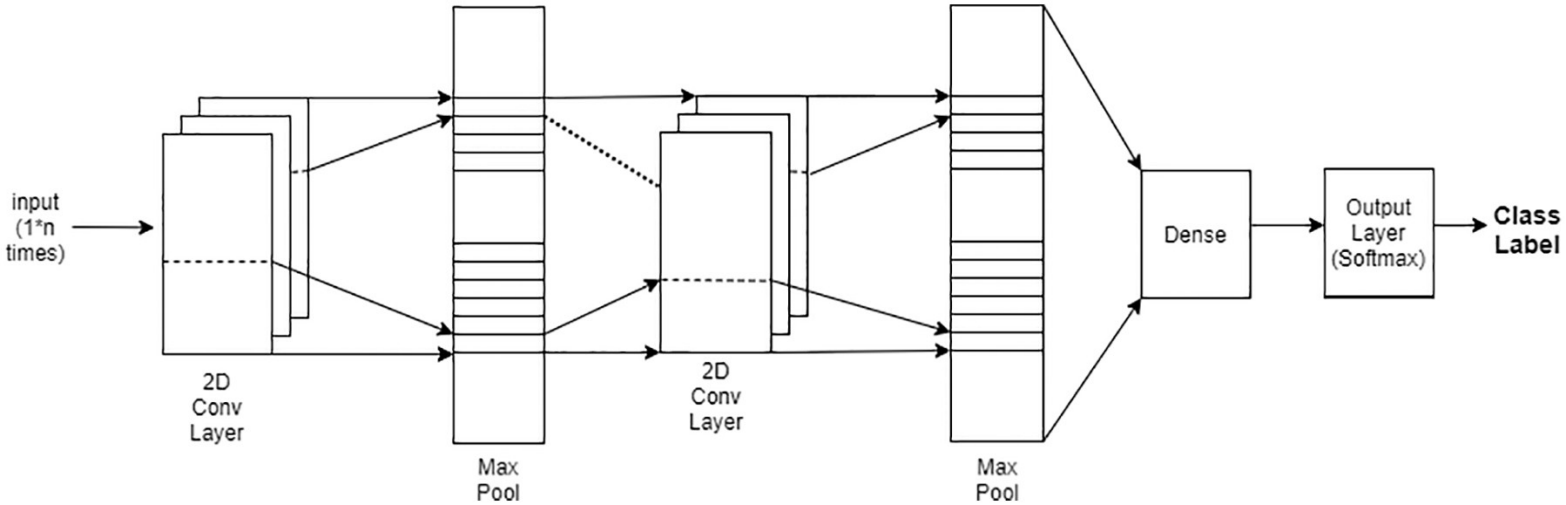
CNN architecture. CNN architecture having two hidden layers, followed by a dense and output layer. The input vector is given to the 1st convolution (hidden) layer, and output is received via the output layer as distribution of softmax function.

Fig 1 provides the overall architecture of our proposed CNN model. Initially, we resize the gene expression as a matrix form to feed into the convolutional layer. The convolutional layers are constructed using two-dimensional kernels that move through the sequence. These kernels act as filters that are being learned during training. As in many CNN architectures, the deeper the layers get, the higher the number of filters becomes. Each convolution layer is followed by pooling layers to reduce the sequence length.

Once the last layer is reached, we need to flatten the tensor and feed it to a classifier with the right number of neurons and therefore use a fully connected layer. Then, the classifier outputs the class of gene expression, and softmax is used as an activation function to provide the output class [43]. We use dropout regularization to prevent over-fitting and use Adam optimizer [44]. We use a batch size of 32 in our training process.

#### Long Short Term Memory (LSTM)

**Fig 2:**
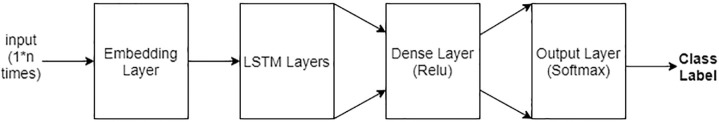
LSTM architecture. LSTM architecture having two hidden layers, followed by a dense and output layer. The input vector is given to the 1st convolution (hidden) layer and output is received via the output layer as distribution of softmax function.

In this method, we resize the gene expressions and feed them into the embedding layer with no initial weights. The features are given weights in this layer and then provided to a basic LSTM layer with 200 cells. Then we add a dense layer with ReLU activation. Finally, softmax is used as an activation function in the output layer [45]. Fig 2 shows the LSTM method architecture.

#### Support Vector Machine (SVM)

Support Vector Machines (SVM) output an optimal hyperplane that categorizes each data point. Given a set of gene expression *D* = {*g*_*i*_}, SVM finds a line/hyperplane that separates the classes. Another important characteristic of the SVM classifier is the margin, which is the separation of a line to the closest data points. For a good margin of the kernel, we have used *L*_2_ loss [46].

#### One-class SVM

One-Class SVM is an unsupervised SVM introduced by Schölkopf et al. [47]. It first maps the data into *d*-dimensional feature space and uses a contour to describe the data embedded in the feature space for each class by putting most of the point of the same class into the contour. If further observations lay within the frontier-delimited subspace, they are considered as coming from the same population as the initial observations. Otherwise, if they lie outside the frontier, they are not considered as the samples from the same class.

For a two-class classification problem, we can learn a binary classifier via conventional models (e.g., logistic regression) to delimit between two class expressions. In the case of *n*-class/label classification problem, we have to learn *n* distinct binary classifiers, each designed for recognizing a particular class. For *d*-dimensional space, each single classifier is a *d* + 1-size kernel, including the intercept term.

#### Deep Neural Network (DNN)

DNN [30] first transforms the raw expression data to categorical state to discover the underlying distinct expression patterns of the generated circadian genes for validating the manually labelled dataset. However, labels of each gene expression are already available for all of our datasets. Therefore, we directly implemented the DNN based on the time series expression data. We employed the same architecture for DNN as [30] with ten hidden layers of 100 nodes. For activation function, the rectified linear unit (ReLU) was used, and the learning rate was set at 0.0001 using an Adam gradient-based optimizer [44]. Dropout layers with a 5% dropout rate were used between each hidden layer to avoid an overfitting problem. The structure of DNN and hyper-parameters were consistent across all datasets.

#### DeepTrust

DeepTrust [31], a convolutional gene clustering framework, initially converts time series data into images to obtain richer data representations. Therefore, we have transformed each gene expression time series to a non-binary image using a global recurrence plot, where patterns on the recurrence plot reflect characteristics of dynamical behaviour. Afterwards, DeepTrust performs a deep convolutional clustering algorithm on the constructed images. The convolutional autoencoder is composed of three connected convolutional layers. The number of filters and kernel sizes of those three layers is set to (32, 64, 128) and (5, 5, 3), respectively, according to the DeepTrust architecture. The dimension of the embedded space was equal to the number of classes in the dataset (e.g., it was 3 for the GSE6186 dataset). The model was trained by Mean Squared Error (MSE) loss and Adam optimizer [44], and the number of epochs was fixed to 200.

### Forecasting

Given a gene *g* and its time series values ${g_{i}}^{T}=\{x_{i_{1}},x_{i_{2}},x_{i_{3}},\ldots ,x_{i_{T}}$} of *T* time periods, forecasting of *k* periods refers to prediction of the values $x_{i_{T+1}},x_{i_{T+2}},\ldots ,x_{i_{T+k}}$ of that time series so that the predicted values conform to the entire series. We provide a detailed description of the forecasting methods included in this study. Similarly to classification, we implement two deep learning based methods LSTM and ANN, whereas Holt-Winters, ARIMA, and Feed Forward Neural Network from GluonTS [41], are used as baseline methods for forecasting in our study.

#### LSTM method

In order to apply LSTM on gene expression prediction, we divide each time series into the training and testing portion. We utilize the training part to fit our model and forecast on the testing portion to evaluate the performance with the predicted values and actual values. A sample pseudo code is provided in Algorithm 1.

**Algorithm 1**: *LSTM*(*X*, *k*)

**Input**: *X* time series of a gene, *k* forecasting period

**Output**: Forecasted series of *k* time period

*X* ← *Normalise*(*X*)

$\bar{X},\bar{y}\leftarrow Supervised\left(X\right)$

*model* = *LSTMmodel*()

$model.fit(\bar{X},\bar{y})$

$history=\{\bar{X}\}$

*prediction* = {}

**for** *i* = 1 → *k* **do**

 *y* ← *model.predict*(*history*)

 *history.add*(*y*)

 *prediction.add*(*y*)

**end**

**return** *prediction*

In order to train the model, we represent the data by using the observation from the last time step (*t* − 1) as the input and the observation at the current time step (*t*) as the output. Then we create an LSTM model with two layers, each having 100 and 50 neurons respectively, and finally, add a dense layer for output. We evaluate the model by optimizing the mean square error (MSE loss) and using Adam [44] optimizer. We set the batch size to 100, and the iteration count to 50. Finally, for *k* forecasting period, we predict the next value based on history. Each predicted value is also added to history.

#### Artificial Neural Network (ANN)

As in LSTM, the time series is divided into training and testing portion, and forecasting is done on testing data. We use the neural network as a multi-layer perceptron regressor since time series can have any value. We convert each gene expression to a vector of *d* dimensions of time values [*x*_*t*−1_, *x*_*t*−2_, …, *x*_*t*−*d*_] and *x*_*t*_ is the output value. Therefore, we use the past *d* values to predict the current values. We add four hidden layers with 5, 10, 15, 20 neurons respectively. We use Sigmoid or Relu as the activation function.

#### Holt-Winters method

Triple Exponential Smoothing, also known as the Holt-Winters method, can be used to forecast data points in a series, provided that the series is “seasonal”, i.e. repetitive over some period [36]. This method calculates a trend line for the data as well as seasonal indices that assign weights to the values in the trend line based on where that time point falls in the cycle of length *L*. Three parameters are configured in the Holt-Winters method.

- **Smoothing coefficient** (*α*) controls the rate at which the influence of the observations at prior time steps decay exponentially.
- **Trend coefficient** (*β*) controls the decay of the influence of the change in trend.
- **Seasonal coefficient** (*γ*) controls the influence on the seasonal component

Moreover, we consider both multiplicative trend and seasonality in forecasting since gene expression does not show any specific one. To forecast a series of season length *L*, we need at least 2*L* historical data. We compute season length *L* from time series representation of a gene. The values of *α*, *β*, *γ* have been fine-tuned during experimentation.

#### ARIMA model

ARIMA (Autoregressive Integrated Moving Average) [35] model is a class of statistical models for analyzing and forecasting time series data. A standard notation *ARIMA*(*p*, *d*, *q*) is used where the parameters are substituted with integer values to quickly indicate the specific ARIMA model. The parameters of the ARIMA model are defined as follows.

- **Lag order (p)**: The number of lag observations included in the model.
- **Degree of differencing (d)**: The number of times where the raw observations are differenced.
- **Order of moving average (q)**: The size of the moving average window.

Those hyperparameters are different for individual datasets, and those were adjusted through tuning.

#### Feed Forward Neural Network

GluonTS [41] provides different deep learning based models for the development and experimentation with time series models for forecasting. We have used GluonTS’s built-in Feed Forward Neural Network [42], a simple but powerful forecasting model as a baseline tool for predicting gene expression. This model includes an input window of length (*context length*) and predicts the distribution of the following *prediction length* values. It can also be configured with different hyperparameters, such as the number of layers, learning rate, epoch numbers. We have used the time period of gene expression (*T*) as the *context length* and forecasting period (*k*) as the *prediction length*. Other hyperparameters were set to default values in all datasets and experimentations.

### Experimental studies

In this subsection, we briefly describe the datasets used in our study and experimental setup for performance evaluation of previously mentioned methods.

#### Dataset description

Gene expression data are usually presented in an expression matrix form. Each column represents all the gene expression levels from a single experiment, and each row represents the expression of a gene across all experiments. Let *G* be the gene expression matrix of size *N* × *M*, where *N* is the total number of genes, and *M* is total time points. *g*_*ij*_ is the log ratio of gene *i* in sample point *j*. The log ratio is defined as $log_{2}\left(\frac{T}{R}\right)$, where *T* is the gene expression level in the testing sample, *R* is the gene expression level in a reference sample. In gene expression experiments, mRNA molecules are typically collected from both an experimental sample and a reference sample. For example, the reference sample could be collected from a healthy individual, and the experimental sample could be collected from an individual with a disease like cancer. Log transformation is used because it is easier to link log ratio to fold change.

We use 5 real datasets in our experiments, and each dataset contains a different number of genes and their associated time values of equal or unequal lengths.

**Fig 3:**
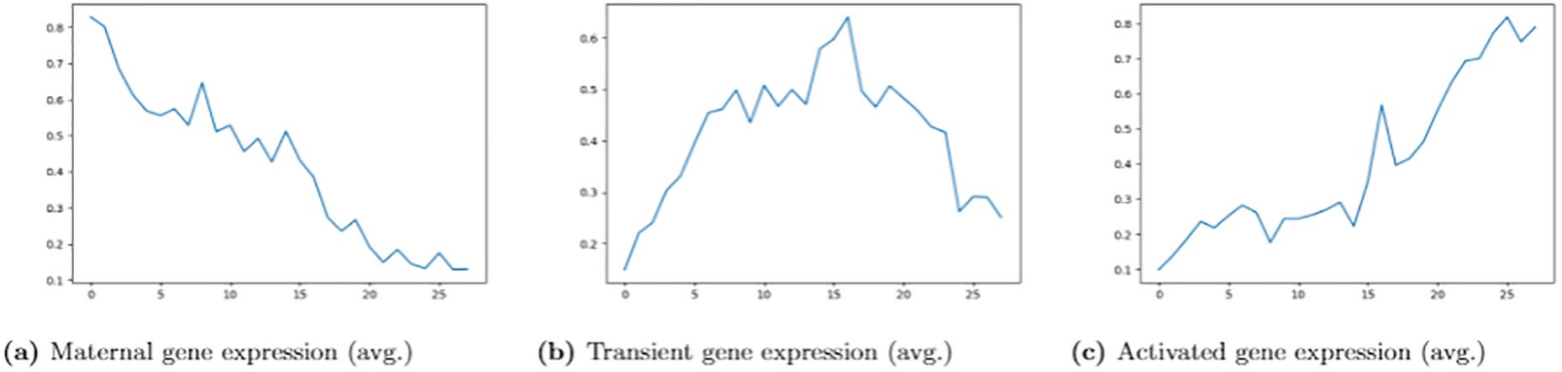
Different classes of GSE6186 gene expression. X-axis denotes the time interval and Y-axis represents the corresponding gene expression value. (A): Maternal gene expression. (B): Transient gene expression. (C): Activated gene expression.

*GSE6186*. The first dataset considered in our tests, denoted as **GSE6186**, was originally described in [48]. It represents whole-genome expression during *Drosophila melanogaster* development. Applying conservative filtering criteria and requiring *sharp* transcript changes, they identified 1534 *maternal* genes, 792 *transient zygotic* genes, and 1053 genes whose transcript levels increase (*activated*) during embryogenesis. Gene expression levels were measured over 24-hours. In particular, measurements were performed at 28 time points with an interval of 0.5 − 1 hour between each pair of recorded values. There are 2943 genes labeled with 3 types of gene (gene label is collected from the supplementary material of [48]). Fig 3 shows sample gene expression of this dataset.

The objective here is to determine the label of a *Drosophila melanogaster* gene as *maternal* or *transient zygotic* or *activated* given its gene expression during embryogenesis. Maternal genes start with a high relative transcript level, which subsequently decreases. Transient gene transcript levels first increase and later decrease, and they do not seem to be initially high like maternal genes. Transcript levels face only one increase in expression for activated genes [48]. The interval for which those 3 categories of gene expression changes is moderately separated.

*GSE3406*. This dataset is referred to as **GSE3406** [49]. It contains gene expression profiles of four yeast species (*S. cerevisiae*, *S. kudriavzevii*, *S. mikatae*, and *S. paradoxus*). The dataset is composed of the expression profiles of 1900 genes. Each gene is perturbed at 5 environmental stresses: heat shock, oxidative stress, growth on glycerol, nitrogen starvation, and DNA damage. For each gene and each medium, expression value is measured at 6 time points from 10 to 90 minutes after the onset of the perturbation. In total, there is 1900 * 4 = 7600 gene expression profiles. Fig 1 in S1 File shows sample gene expression of this dataset. In this case, the goal is to distinguish 4 closely related yeast species given their gene expression under 5 different stress level. Transcript level for different species at the same stress was typically more correlated than the same species at different stresses [49]. Perhaps, thus, there is no clear differentiation in Fig 2 in S1 File.

*GSE1723*. Another dataset considered in our study is referred to as **GSE1723** [50]. It contains gene expression profiles of transcriptional responses at 4 different nutrient-limitation regimes (viz, carbon, nitrogen, sulphur, phosphorus) under *aerobic* and *anaerobic* conditions in chemostat cultures of *S. cerevisiae*. The dataset is composed by the expression profiles of 9326 genes and 9326 * 2 = 18652 gene expression profiles in total. Fig 2 in S1 File shows sample gene expression of this dataset. For this dataset, we want to predict the environment (viz, aerobic, or anaerobic) of a given gene, based on the expressions in different conditions. In the dataset, 42% of gene expression does not show any transcript change across all eight conditions. Only, 2.6% (155) of gene expression is consistently responsive to oxygen [50].

*Patient*. The next dataset, indicated as **MS**–**rIFNβ** or **patient** data in short, was first analyzed in [51]. It contains gene expression profiles of 52 patients who have relapsing-remitting multiple sclerosis (MS). They are classified as either good (33) or poor (19) responders to recombinant human interferon-beta (rIFN*β*). The dataset is composed of the expression profiles of 70 genes isolated from each patient at 7 time points: before the administration of the first dose of the drug (*t* = 0), every 3 months (*t* = 1, 2, 3, 4) and every 6 months (*t* = 5, 6) in the first and second year of the therapy, respectively. For a few patients, entire profile measurements are missing at 1 or 2 time points. We consider gene from each patient as a separate identity, and therefore, we get 3640 gene expressions in total. We make each expression of an equal length of nine by fitting the missing values with interpolation. We normalize the expression to prepare them for the model. Fig 3 in S1 File shows sample gene expression for good and bad responders in this dataset. The objective is to determine whether a patient is a good/bad responder to interferon beta, given one’s 70 gene expressions. Interferon beta has a relatively large proportion of non-responders. Moreover, no reliable separation can be drawn concerning their response status by applying similarity/dissimilarity measures and clustering algorithms. The difference between the two groups is also subtle by conventional measures [51].

*Yeast*. The final dataset considered in our experiments, denoted as **Yeast**, was originally described in [52]. It contains the genome characterization of the mRNA transcript levels during the cell cycle of the yeast *Saccharomyces cerevisiae*. Gene expression levels were gathered at regular intervals during the cell cycle. In particular, measurements were performed at 17 time points with an interval of 10 minutes between each pair of recorded values. Different experiments are done in the gene expression time series of this dataset are known to be associated to 5 different phases, namely Early G1/M, G1, S, G2 and M which represent the class values in their setting. From the dataset, 87 genes are labeled with these distinct phases. Therefore, we only consider these gene expressions in our problem. Fig 4 in S1 File shows sample gene expression for various phases in this dataset. The classification target is to predict the genes associated with different phases of mitosis cell division from given gene expressions. The dataset comprises of 2 complete cell cycle. Transcript changes showed a good harmony between the two-time courses. Only 7% of yeast genes were identified that demonstrated consistent periodic changes in transcript level. Moreover, some transcripts were affected by more than one cell cycle-dependent regulatory sequence, and some genes were expressed in two different cell cycle phases [52].

Among the datasets, patient and yeast datasets are imbalanced. In the patient dataset, the number of good responders is twice the number of bad responders. In the yeast dataset, almost 50% expression is of G1 phase, and less than 10% expression is of G2 phase.

#### Running STEM

In addition to the classification methods discussed earlier, we have used the STEM clustering method [15] to assess the performance of a clustering algorithm in classification tasks. We have set the maximum number of model profiles to the actual number of class labels in the dataset (e.g., for the GSE6186 dataset, this parameter was set to 3 as there are 3 different types of genes). After executing, we have got a different gene for each profile.

In each gene profile, we have calculated the number of different class labels. We have constructed a matrix where each row represents a gene profile for all gene profiles, and each column represents a class label. Then after row and column swapping, we transform it into a confusion matrix. The objective of the swapping was to maximize the diagonal sum.

#### Evaluation criteria

*Classification*. We have considered accuracy and F1 score (from confusion matrix) as the performance metric for classification. *Accuracy* for a dataset can be defined as,

$$\begin{array}{c} Accuracy=\frac{\#\text{correctly}\;\text{classified}\;\text{gene}\;\text{expressions}}{\#\text{gene}\;\text{expressions}} \end{array}$$

To compute *F1 score*, we have calculated *precision* and *recall* for each label and finally averaged them. Moreover, we have compared our results on classification with a baseline clustering method [15]. For this purpose, we have performed clustering of gene expression for each dataset using the STEM [16] software, as discussed above.

*Forecasting*. Similarly, to evaluate the performance of described forecasting techniques, we have used Root Mean Squared Error (*RMSE*). Let *x*_1_, *x*_2_, …, *x*_*k*_ be actual values of a time series *X* and forecasting values for *k* periods are measured as $x_{1}^{\prime },x_{2}^{\prime },\ldots ,x_{k}^{\prime }$, then RMSE error is defined as,

$$\begin{array}{c} RMSE\left(X\right)=\sqrt{\sum_{i=1}^{k}\frac{{\left(x_{i}-x_{i}^{\prime }\right)}^{2}}{k}} \end{array}$$

#### Experimental setup

All of our experiments are implemented in Python. Moreover, we use external packages and libraries for various methods. Supervised and unsupervised machine learning-based models for classification have been implemented with Python machine learning packages scikit-learn (http://scikit-learn.org/stable/). We use Statsmodels (http://www.statsmodels.org/stable/index.html) for implementing the ARIMA model in gene expression forecasting. For deep-learning-based LSTM and Neural Network method, we use the Python Deep Learning library Keras (https://keras.io/) using Tensorflow (https://www.tensorflow.org/) as backend.

Experimental evaluation was conducted on a machine with an Intel Core i7 processor with 2.5GHz clock speed and 16GB RAM. The machine also has an Nvidia GTX 960M with 4GB memory and therefore Tensorflow based experiments can utilize GPU instructions. All of our codes and data are available publicly (https://github.com/mahi045/time-series-gene-expression.git).

## Results

In this section, we provide a detailed analysis of classification and forecasting results and the relative performance of various approaches on each dataset. We also discuss which methods can be further developed and tailored for gene expression time series analysis.

### Classification results

Tables 1 and 2 show the performance of various classification method on our datasets. For CNN, DNN, and LSTM methods, we have used 70%, 10%, and 20% gene expressions as train, validation, and test set, respectively. Training and testing percentages are 80, and 20, respectively, for both SVMs. In both tables, each row represents the statistics of a dataset, and each column represents the statistics of a specific method.

**Table 1:** Accuracy of all methods on different datasets. All accuracy value is mentioned in percentage (%).

Dataset	Accuracy					
	CNN	LSTM	SVM	One-Class SVM	DNN	DeepTrust
GSE6186	**96.15**	92.19	95.75	93.02	93.21	78.23
GSE3406	**93.14**	86.38	88.83	50.13	87.36	43.28
GSE1723	80.25	76.15	83.11	73.54	**84.88**	59.3
Patient	**82.04**	64.21	68.14	63.45	63.07	53.55
Yeast	**88.42**	47.37	63.17	47.36	78.94	55.56

**Table 2:** F1 score of all methods on different datasets. All F1 score value is mentioned in percentage (%).

Dataset	F1 score					
	CNN	LSTM	SVM	One-Class SVM	DNN	DeepTrust
GSE6186	**94.23**	92.33	93.59	92	93.18	39.18
GSE3406	85.86	86.45	**90.92**	50.1	87.1	16.46
GSE1723	80.25	76.15	83.03	73.54	**84.85**	18.61
Patient	**64.09**	63.71	57.86	46.13	63.02	18.63
Yeast	**66.67**	47.37	63.17	30	53.84	17.78

In each dataset, CNN, SVM, or DNN has the best performance among all the methods. On the other hand, the accuracy of the one-class SVM or DeepTrust is relatively low, and LSTM shows intermediate performance. All methods perform well on the GSE6186 dataset. One possible explanation is that all expressions of the same phase follow a particular pattern (Fig 3). On the GSE3406 dataset, CNN, LSTM, and SVM demonstrated satisfactory performance. Correlations between gene expressions of four species may have led to the poor performance of the one-class SVM. Compared to the first two datasets, most of the methods have lower accuracy and F1 scores on the GSE1723 dataset. In the GSE1723 dataset, several gene expressions are oxygen irresponsive, both aerobic-anaerobic responsive, and of complex nature [50]. It could be the primary reason for the relatively bad performance of all methods. CNN has performed better than other methods in both patient and yeast datasets. The difference between good and bad responders is exact in the patient dataset. In the yeast dataset, a high portion of the genes does not display consistently periodic change in the transcript. Possibly, that is why all methods have generally failed to differentiate them. Moreover, both patient and yeast datasets are imbalanced too. Probably, for this reason, the F1 score is sometimes significantly lower than the accuracy in these two datasets. The performance of LSTM is less satisfactory than CNN, DNN, and SVM. One possible explanation may be that gene expression goes through different environments (e.g., there are four nutrient-limited regimes in GSE1723 and five perturbations in GSE3406), and it is not wise to include previous time point’s expression value. Finally, we can conclude that machine learning (e.g., SVM) and deep learning (e.g., CNN, DNN) approaches can be further developed to improve the classification of microarray time series gene expression. The poor performance of DeepTrust implies that extending time series to image for information gain is not suitable for time series gene expression classification. Neural network architecture on raw gene expression is satisfactory for gene expression classification, even if there are limited time points.

To compare our methods against clustering, we have run all the datasets on STEM. The result is presented in Table 3. In the table, each column represents performance on a dataset. On all datasets, the performance of STEM is worse than classification methods, sometimes substantially so. Therefore, we can conclude that clustering has not come up with satisfactory insights that classification has brought to a great extent.

**Table 3:** Accuracy & F1 score of STEM on all dataset. All value is mentioned in percentage (%).

	GSE6186	GSE3406	GSE1723	Patient	Yeast
Accuracy	75.5	30.45	51.64	52.12	42.55
F1 score	61.4	31.19	51.64	50.83	25.38

### Forecasting results

We have implemented Artificial neural network, LSTM for forecasting, and compared the performance with Holt-Winters, ARIMA, and Feed Forward Neural Network [42] from GluonTS toolkit. We divide the dataset into train and test set to build the model based on training data and compute forecasting on the rest of the portion to compare with the actual test data. We use 10%, 20%, 30%, and 40% of a time series as testing data. Table 4 shows RMSE error of different methods in all datasets. In the table, each row represents statistics of a dataset for a specific test percentage. The performance of various methods in forecasting was assessed based on the following criteria.

- Overall performance of each method on various datasets
- Performance of various methods with varying amounts of test data
- Quality of prediction

It is worth noting that there are several cases where some of our forecasting methods could not be executed (denoted by ‘-’ in Table 4). It happens either due to insufficient training time points, failure to initialize several parameters, or inadequate testing time points for evaluation. Moreover, the series is too short in some cases, and we failed to run several methods on them. However, in all cases, either Holt-Winters or LSTM has the maximum error, and ARIMA or ANN performs the best. Gene expression experiments often go through different environments, and sometimes the corresponding time interval is not equal. Therefore, it is perhaps unjustified to include previous time points’ expression value (like LSTM), assumption of seasonality, trend line (like Holt-Winters), or context length (like Feed Forward Neural Network) during forecasting.

**Table 4:** RMSE value of all methods on different datasets. RMSE value of different methods for different test percents are grouped together and best RMSE values are highlighted.

Test percent	Method	GSE 6186	GSE 3406	GSE 1723	Patient	Yeast
10	Holt-Winters	0.3	0.52	0.53	-	0.48
	ARIMA	**0.244**	**0.5**	**0.35**	-	**0.37**
	ANN	0.268	0.55	0.54	-	0.5
	LSTM	0.3	0.76	-	-	-
	GluonTS	0.361	0.76	0.831	-	0.599
20	Holt-Winters	0.538	0.7	0.72	0.8	0.79
	ARIMA	**0.338**	**0.63**	0.54	0.414	0.57
	ANN	0.5	0.65	0.65	**0.33**	0.64
	LSTM	0.6	0.9	**0.53**	-	**0.45**
	GluonTS	0.561	0.798	1.02	0.835	0.874
30	Holt-Winters	0.665	0.87	0.93	0.94	1.13
	ARIMA	**0.362**	**0.63**	**0.64**	**0.442**	0.64
	ANN	0.488	**0.63**	0.67	-	0.56
	LSTM	0.746	0.86	0.73	1.1	**0.53**
	GluonTS	0.725	0.985	1.161	1.066	1.4
40	Holt-Winters	0.8	1.1	1.07	1.2	1.31
	ARIMA	-	-	-	-	-
	ANN	**0.705**	**0.6**	**0.65**	-	**0.53**
	LSTM	0.88	0.91	0.88	1.35	0.59
	GluonTS	1.28	1.29	1.90	2.2	1.93

For Holt-Winters, ARIMA, and Feed Forward Neural Network method, RMSE error increases almost proportionately with an increasing amount of test data. However, for ANN and LSTM, RMSE error does not increase in proportion to the test data percentage, even decreases in some datasets. Therefore, we can conclude that deep learning based methods with back propagation or recurrent cells in the architecture might be suitable for long time series prediction.

In order to visualize forecasting in some scenarios, we have presented several figures (Figs 5-21) in S1 File by superimposing actual and predicted gene expression on different datasets with varying amount of test data. From those figures, we observe that the forecasting methods can capture trends in gene expression data and can be used for forecasting in this domain. Moreover, from Table 4, it is quite evident that ARIMA can work well when more expression values are available. ANN can learn from the existing expression values and perform better in predicting longer sequences. However, multi layer perceptron architecture from the Feed Forward Neural Network method without back propagation or additional cells to remember the sequence of data from the LSTM method is not suitable in forecasting in the gene expression domain.

In summary, we observe that ARIMA and ANN have the best performances among the five methods we considered where ARIMA is the best suited for short term forecasting and ANN is better suited for long term forecasting. Therefore, we recommend ARIMA and ANN for time series gene expression forecasting and suggest further tailoring by considering unique patterns and attributes in gene expression data.

## Discussion

In this study, we have proposed and investigated different classification and forecasting methods for time series gene expression data. To verify the efficiency and effectiveness of these methods; we have conducted an extensive experimental study on five real gene expression datasets, and compared state-of-the-art techniques along with methods proposed in this paper. We find that a CNN based architecture presented here generally outperforms other methods for gene expression time series classification, whereas ARIMA and ANN are the best suited for forecasting purposes.

Although our study demonstrates the power and efficacy of various ML and deep learning based methods in gene expression classification and forecasting, this study is limited in scope and can be extended in several directions. Most of the available time series gene expression datasets are fairly short. Therefore, we only propose simple CNN and LSTM model (with only one hidden layer and straightforward architecture) for short time series classification. From Table 4, it is noticeable that the proposed techniques failed to calculate the forecasted value on some short time series instances. Moreover, these datasets often contain some anomalous gene expressions. For example, the GSE1723 dataset contains some gene expressions that respond to both aerobic and anaerobic conditions or complex functionality [50]. Yeast dataset includes some transcripts that might be affected by more than one cell cycle-dependent regulatory sequence), which could be a weakness in our analysis [52]. We have normalized the gene expressions to avoid an extensive range of values. Therefore, the transformed time series might not capture the actual gene expression in approaches.

We can focus on anomaly detection for gene expression, gene expression concurrence, and approximate gene classification as future directions of our work. Any gene expression might be considered anomalous if the corresponding time series significantly deviates from the usual expressions. Here, usual gene expression and degree of deviation will be estimated empirically. In this study, we have found that some gene expressions follow the same pattern, i.e., they might be called correlated. Gene expression concurrence finds a set of genes that most likely changes their expression value concomitantly. Therefore, we can empirically estimate set size, degree of correlation from this gene expression concurrence. For example, the Patient dataset can be effectively classified if there are some other small size clusters [51]. From this observation, we can assume that approximate expression classification will skip the classification of non-conforming gene expression.

Despite the limitations, we can assert that some classification methods have effectively grouped gene expression according to their label, where short time series clustering might not perform well. Besides, extending short time series to images does not enhance classification performance. deep learning based models with simple architecture can effectively classify short time series gene expressions that have specific patterns. Moreover, several forecasting techniques can adjust the gene expression time series efficiently to predict future values. ANN can be particularly useful for long time series expressions where ARIMA provides overall better performance. The timing of this study seems appropriate as gene expression data are becoming more accessible and less expensive. Gene expression analyses are also getting significant attention from the research community in various applications including healthcare. Finally, we are witnessing a rapid acceleration in the use of various ML and deep learning based methods for analyzing time series data. Thus, this study advances the state-of-the-art in time series gene expression analysis, and lays a firm, broad foundation for the application of gene expression classification and forecasting.

## Supporting information

S1 File (PDF)
