# Supplementary material for: Evaluation of classification and forecasting methods on time series gene expression data
Source: PLoS One. 2020 Nov 6;15(11):e0241686. doi: 10.1371/journal.pone.0241686 (PMC7647064; doi:10.1371/journal.pone.0241686)
Supplement: S1 File — (PDF) [file pone.0241686.s001.pdf]

Evaluation of classification and forecasting methods on time series gene expression data : Supplementary Material

October 3, 2020

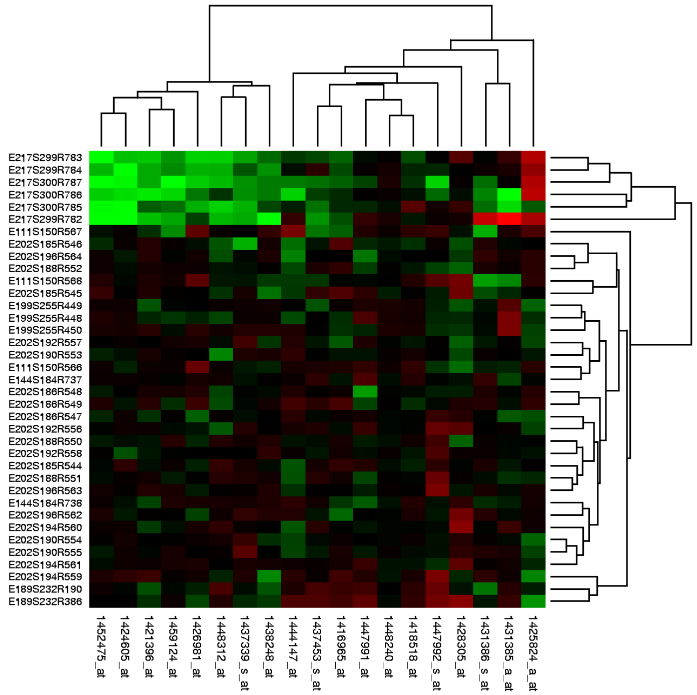

S1 Sample gene expression of different dataset

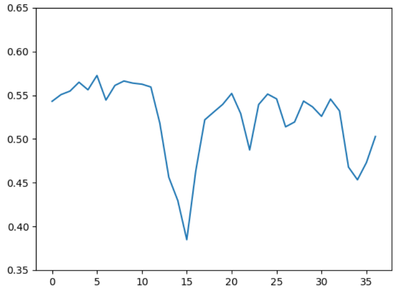

S. cerevisiae

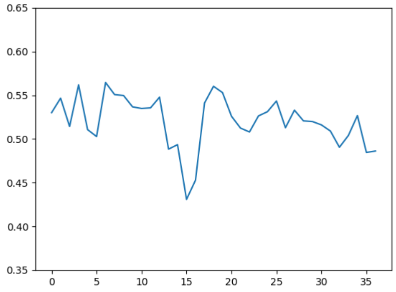

S. kudriavzevii

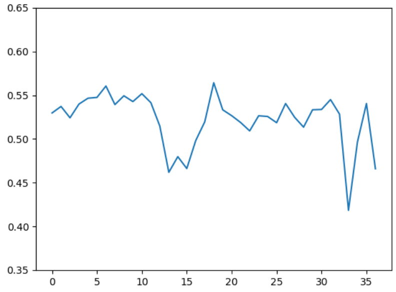

S. mikatae

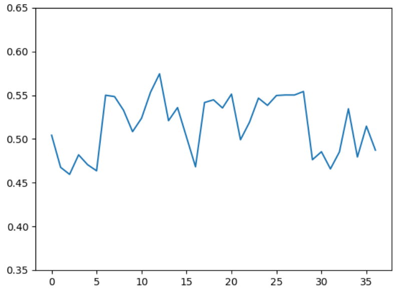

S. paradoxus

Figure 1: **Different species of GSE3406 gene expression.** X-axis denotes the time interval and Y-axis represents the corresponding gene expression value.

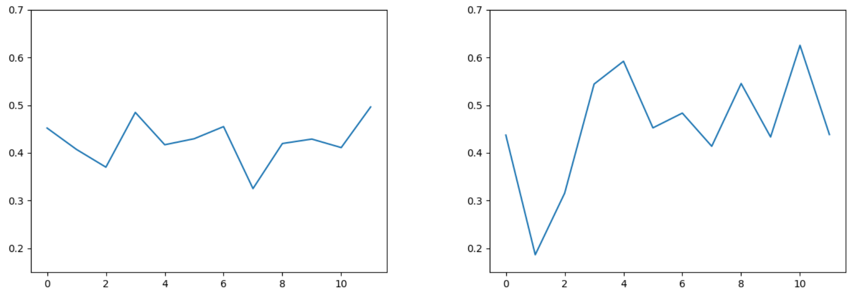

Aerobic phase

Anaerobic phase

Figure 2: **Sample Gene expression from GSE1723 dataset.** X-axis denotes the time interval and Y-axis represents the corresponding gene expression value.

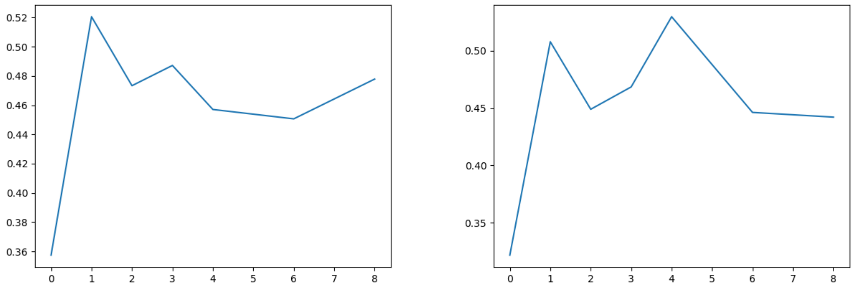

Good responder

Bad responder

Figure 3: **Sample Gene expression from Patient dataset.** X-axis denotes the time interval and Y-axis represents the corresponding gene expression value.

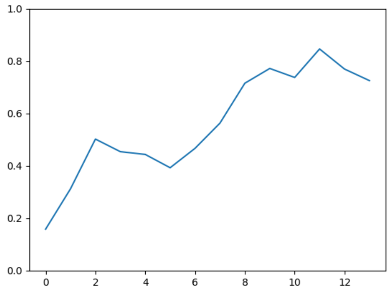

G1 phase

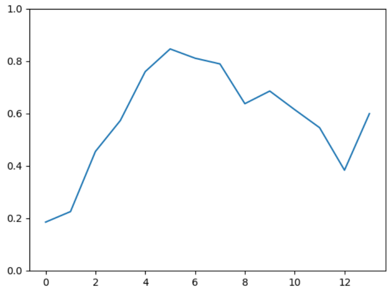

G2 phase

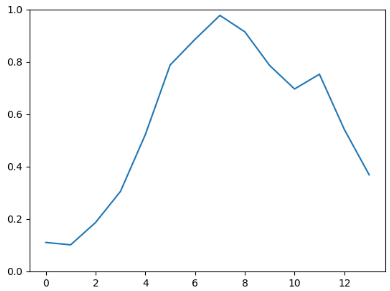

M phase

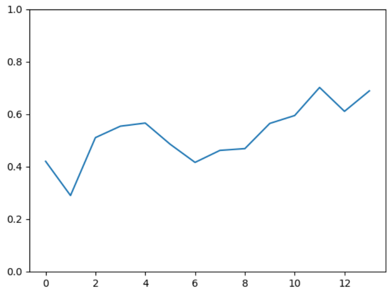

S phase

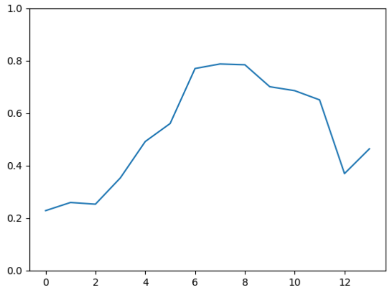

M/G1 phase

Figure 4: **Different phase of yeast gene expression.** X-axis denotes the time interval and Y-axis represents the corresponding gene expression value.

S2 Visual comparison of actual and forecasted gene expression

The following figures (Fig 5 – 16) are showing the visual comparison between actual gene expression and predicated gene expression. For each dataset and specific test percentage, we ran forecasting methods on certain number (viz, 100) of gene expressions . In the following plots, we are using the following short forms:

avg. RMSE error: Pictorial gene expression where RMSE error is median among all RMSE errors.

min. RMSE error: Pictorial gene expression where RMSE error is minimum among all RMSE errors.

max. RMSE error: Pictorial gene expression where RMSE error is maximum among all RMSE errors.

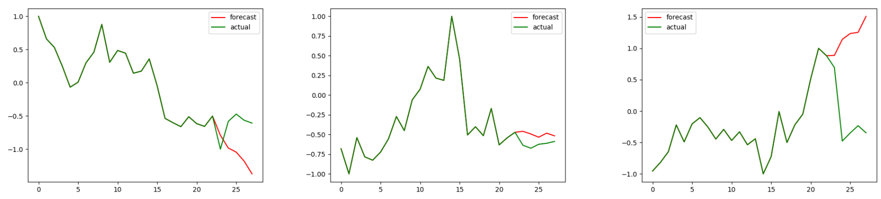

avg. RMSE error   min. RMSE error   max. RMSE error

Figure 5: Forecasted output and actual value on GSE6186 dataset, holtz-winters method with 20% test percentage

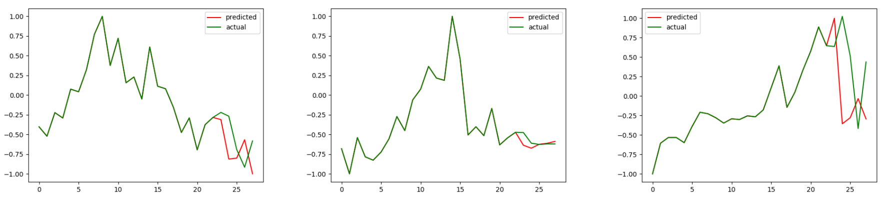

avg. RMSE error   min. RMSE error   max. RMSE error

Figure 6: Forecasted output and actual value on GSE6186 dataset, ARIMA method with 20% test percentage

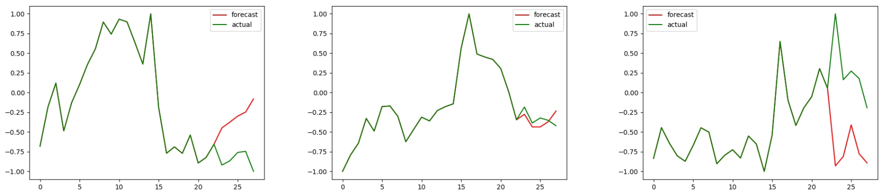

avg. RMSE error | min. RMSE error | max. RMSE error

Figure 7: Forecasted output and actual value on GSE6186 dataset, ANN method with 20% test percentage

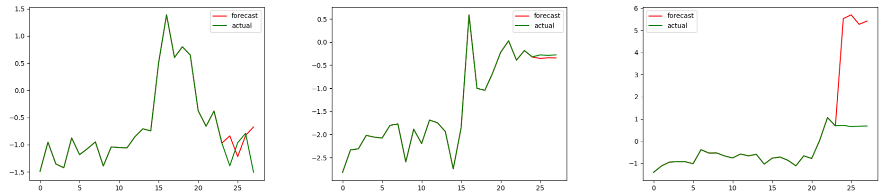

avg. RMSE error | min. RMSE error | max. RMSE error

Figure 8: Forecasted output and actual value on GSE6186 dataset, LSTM method with 20% test percentage

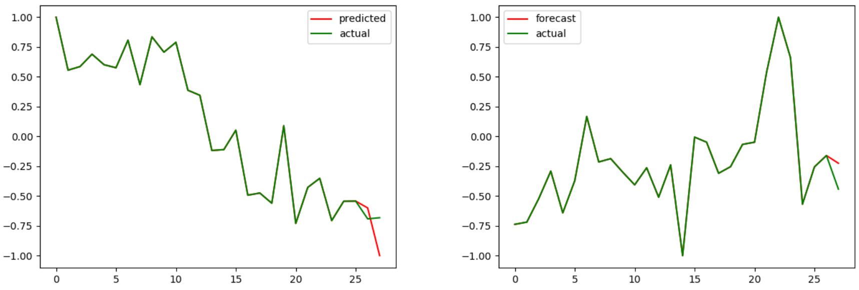

Arima Method | LSTM Method

Figure 9: Forecasted output and actual value on GSE6186 dataset with 10% test percentage

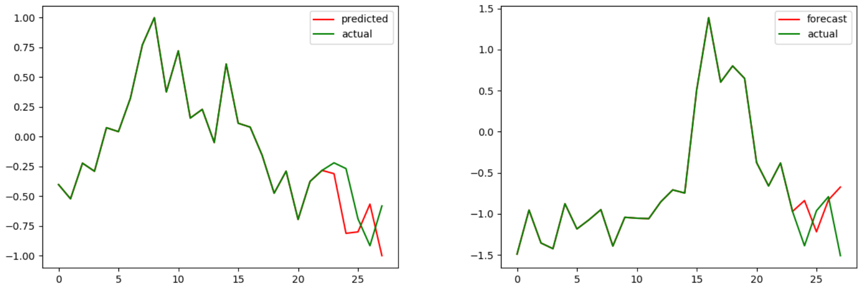

Arima Method LSTM Method

Figure 10: Forecasted output and actual value on GSE6186 dataset with 20% test percentage

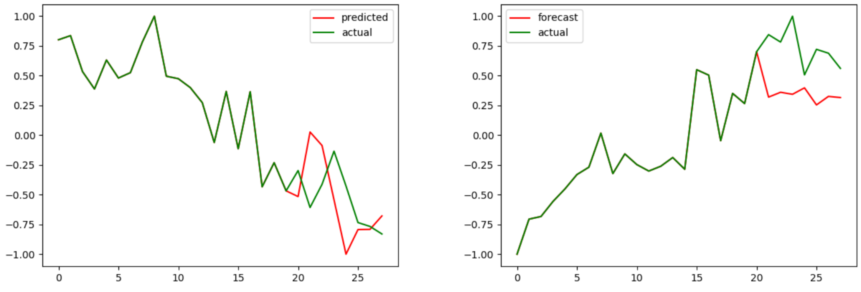

Arima Method LSTM Method

Figure 11: Forecasted output and actual value on GSE6186 dataset with 30% test percentage

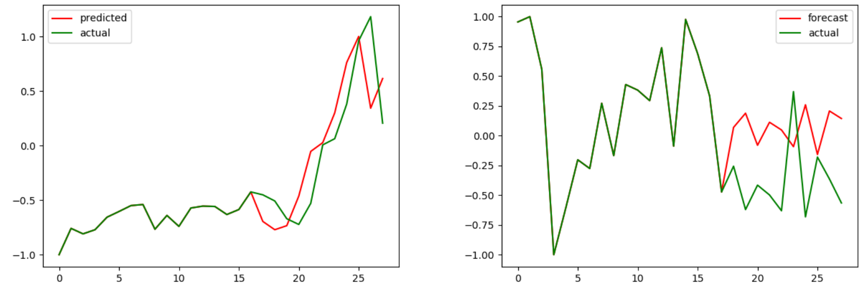

Arima Method LSTM Method

Figure 12: Forecasted output and actual value on GSE6186 dataset with 40% test percentage

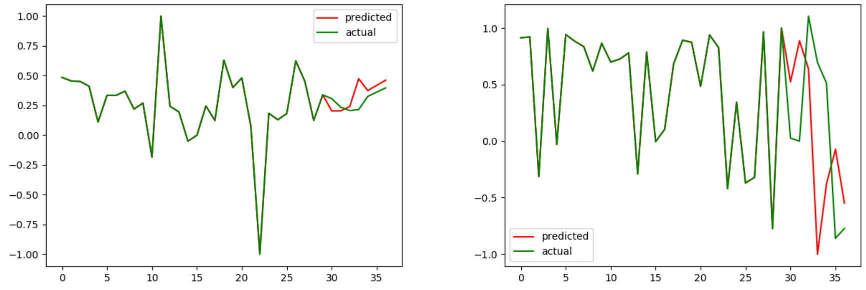

min. RMSE error (Arima) max. RMSE error (Arima)

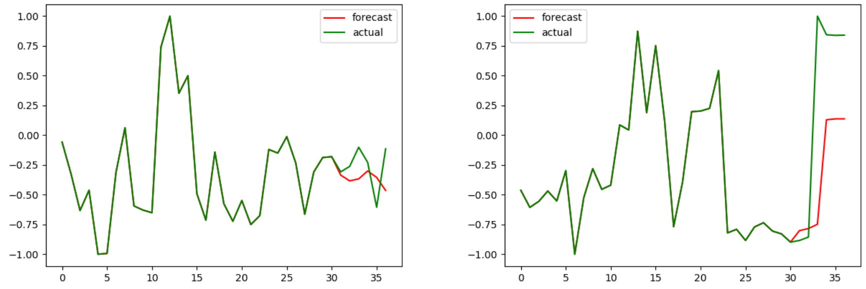

min. RMSE error (LSTM) max. RMSE error (LSTM)

Figure 13: Forecasted output and actual value on GSE3406 dataset with 20% test percentage

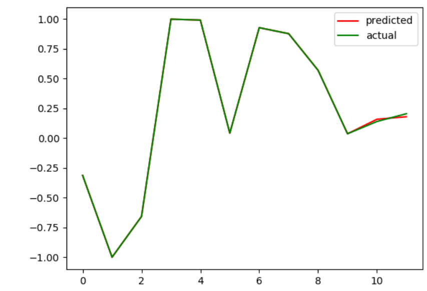

min. RMSE error (Arima)

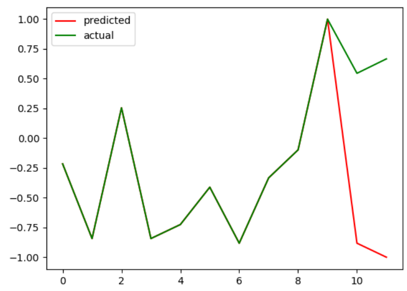

max. RMSE error (Arima)

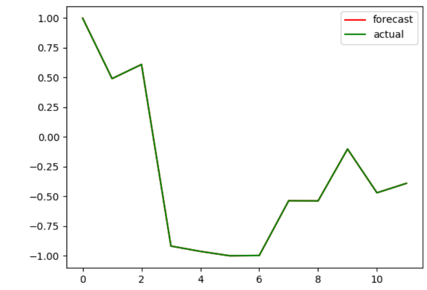

min. RMSE error (LSTM)

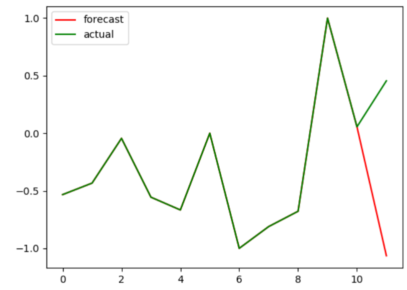

max. RMSE error (LSTM)

Figure 14: Forecasted output and actual value on GSE1723 dataset with 20% test percentage

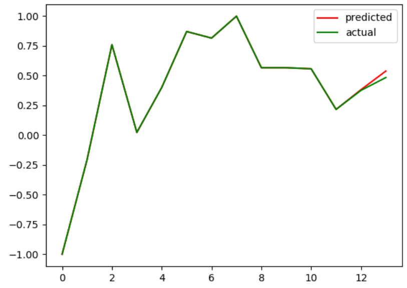

min. RMSE error (Arima)

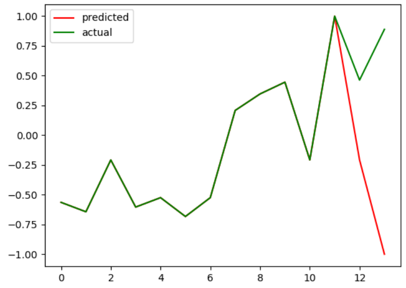

max. RMSE error (Arima)

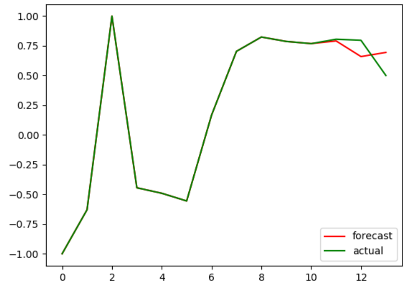

min. RMSE error (LSTM)

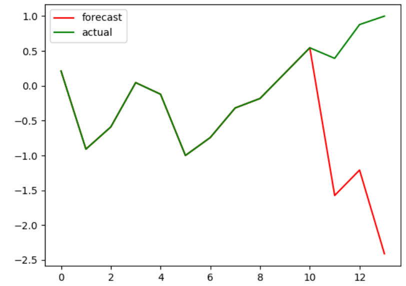

max. RMSE error (LSTM)

Figure 15: Forecasted output and actual value on Yeast dataset with 20% test percentage

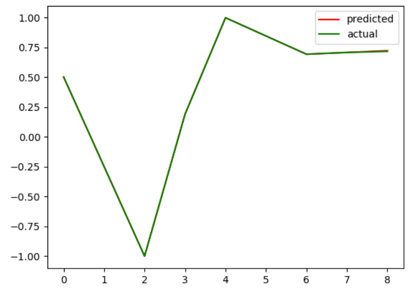

min. RMSE error (Arima)

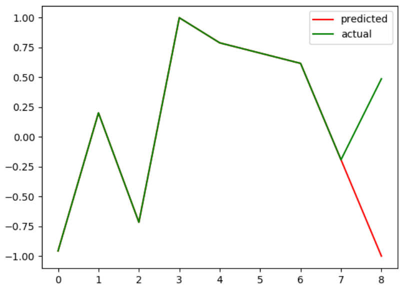

max. RMSE error (Arima)

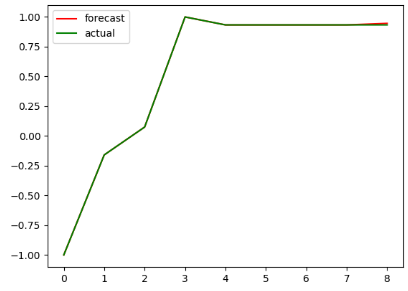

min. RMSE error (LSTM)

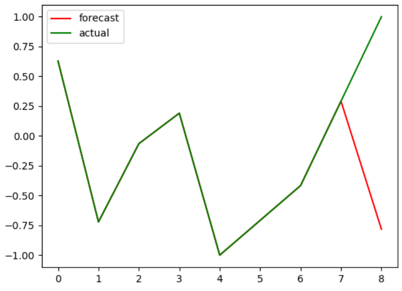

max. RMSE error (LSTM)

Figure 16: Forecasted output and actual value on Patient dataset with 20% test percentage

S3 `GluonTS` visual comparison of actual and forecasted gene expression

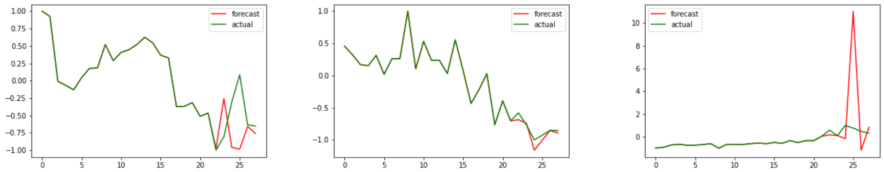

(a) avg. RMSE error (b) min. RMSE error (c) max. RMSE error

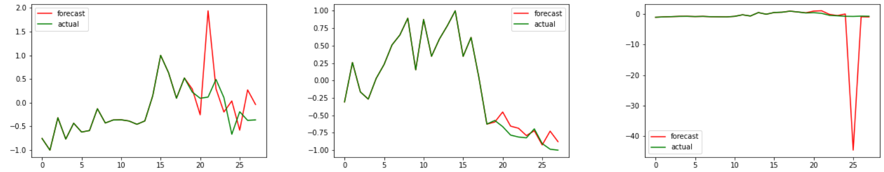

(d) avg. RMSE error (e) min. RMSE error (f) max. RMSE error

Figure 17: GluonTS forecasted output and actual value on GSE6186 dataset with (a) - (c) 20% and (d) - (f) 30% test percentage

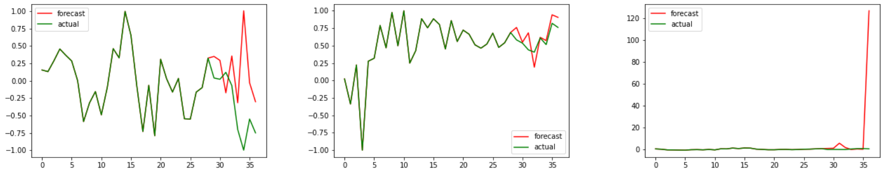

(a) avg. RMSE error (b) min. RMSE error (c) max. RMSE error

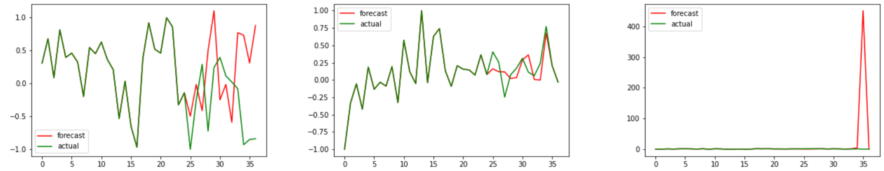

(d) avg. RMSE error (e) min. RMSE error (f) max. RMSE error

Figure 18: GluonTS forecasted output and actual value on GSE3406 dataset with (a) - (c) 20% and (d) - (f) 30% test percentage

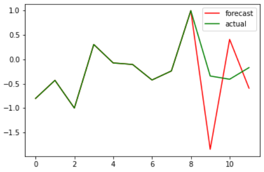

(a) avg. RMSE error

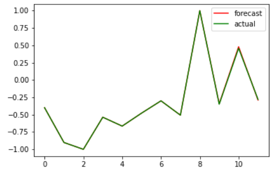

(b) min. RMSE error

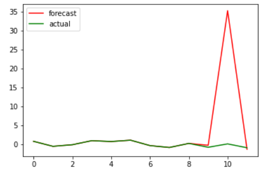

(c) max. RMSE error

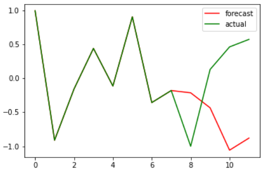

(d) avg. RMSE error

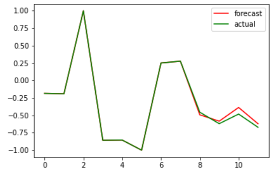

(e) min. RMSE error

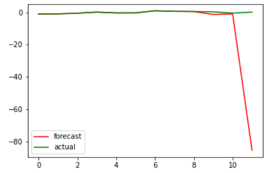

(f) max. RMSE error

Figure 19: GluonTS forecasted output and actual value on GSE1723 dataset with (a) - (c) 20% and (d) - (f) 30% test percentage

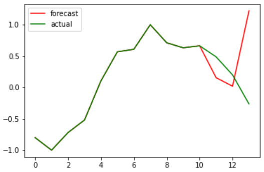

(a) avg. RMSE error

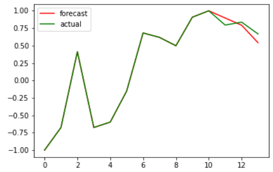

(b) min. RMSE error

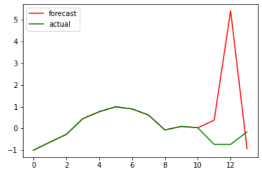

(c) max. RMSE error

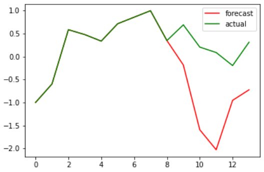

(d) avg. RMSE error

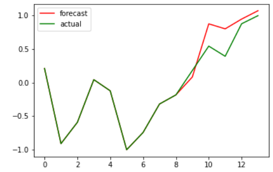

(e) min. RMSE error

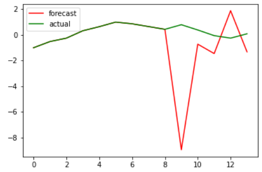

(f) max. RMSE error

Figure 20: GluonTS forecasted output and actual value on yeast dataset with (a) - (c) 20% and (d) - (f) 30% test percentage

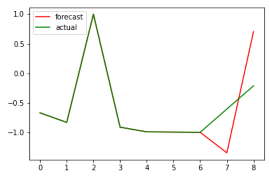

(a) avg. RMSE error

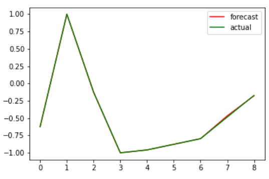

(b) min. RMSE error

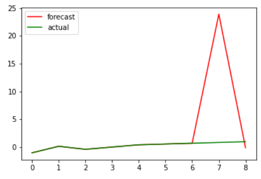

(c) max. RMSE error

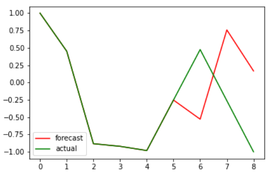

(d) avg. RMSE error

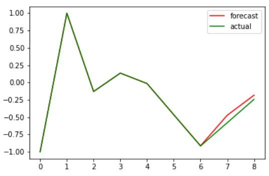

(e) min. RMSE error

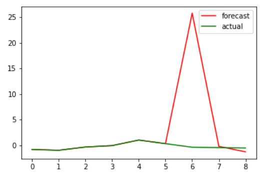

(f) max. RMSE error

Figure 21: GluonTS forecasted output and actual value on yeast dataset with (a) - (c) 20% and (d) - (f) 30% test percentage
